# Clinical impact of manual scoring of peripheral arterial tonometry in patients with sleep apnea

**DOI:** 10.1007/s11325-021-02531-9

**Published:** 2022-04-02

**Authors:** Samuel Tschopp, Urs Borner, Wilhelm Wimmer, Marco Caversaccio, Kurt Tschopp

**Affiliations:** 1 Department of Otorhinolaryngology, Head and Neck Surgery, Inselspital, University Hospital and University of Bern, Freiburgstrasse 18, 3010 Bern, Switzerland; 2 Department of Otorhinolaryngology, Head and Neck Surgery, Kantonsspital Baselland, Liestal, Switzerland; 3 Hearing Research Laboratory, ARTORG Center for Biomedical Engineering Research, University of Bern, Bern, Switzerland

**Keywords:** Sleep apnea, Obstructive, Sleep study, Peripheral arterial tonometry, Home sleep apnea testing

## Abstract

**Purpose:**

The objective was to analyze the clinical implications of manual scoring of sleep studies using peripheral arterial tonometry (PAT) and to compare the manual and automated scoring algorithms.

**Methods:**

Patients with suspected sleep-disordered breathing underwent sleep studies using PAT. The recordings were analyzed using a validated automated computer-based scoring and a novel manual scoring algorithm. The two methods were compared regarding sleep stages and respiratory events.

**Results:**

Recordings of 130 patients were compared. The sleep stages and time were not significantly different between the scoring methods. PAT-derived apnea-hypopnea index (pAHI) was on average 8.4 events/h lower in the manually scored data (27.5±17.4/h vs.19.1±15.2/h, *p*<0.001). The OSA severity classification decreased in 66 (51%) of 130 recordings. A similar effect was found for the PAT-derived respiratory disturbance index with a reduction from 31.2±16.5/h to 21.7±14.4/h (*p*<0.001), for automated and manual scoring, respectively. A lower pAHI for manual scoring was found in all body positions and sleep stages and was independent of gender and body mass index. The absolute difference of pAHI increased with sleep apnea severity, while the relative difference decreased. Pearson’s correlation coefficient between pAHI and oxygen desaturation index (ODI) significantly improved from 0.89 to 0.94 with manual scoring (*p*<0.001).

**Conclusions:**

Manual scoring results in a lower pAHI while improving the correlation to ODI. With manual scoring, the OSA category decreases in a clinically relevant proportion of patients. Sleep stages and time do not change significantly with manual scoring. In the authors’ opinion, manual oversight is recommended if clinical decisions are likely to change.

**Supplementary Information:**

The online version contains supplementary material available at 10.1007/s11325-021-02531-9.

## Introduction

Obstructive sleep apnea (OSA) is characterized by repetitive collapse of the upper airway resulting in arousals and sleep fragmentation [1]. This leads to disturbances in many biological processes and is associated with a higher risk for hypertension, heart failure, stroke, diabetes, and other diseases [2–4]. The prevalence of OSA, defined by an apnea-hypopnea index (AHI) greater than 15/h, is estimated to be as high as 23.4% in women and 49% in men [5]. Due to its high prevalence and associated complications, OSA has major socioeconomic relevance. However, it is believed that 93% of women and 83% of men with OSA remain undiagnosed and thus untreated [6]. Therefore, it is important to offer a cost-effective and reliable diagnosis of OSA.

Currently, sleep laboratory testing using polysomnography is the gold standard for diagnosing OSA. However, home sleep apnea testing (HSAT) offers the possibility for cost-effective and accurate assessment of OSA in selected patients [7]. It is less resource intensive and allows assessment of a patient’s sleep in his habitual sleeping environment. Furthermore, multiple-night testing can be performed to reduce the night-to-night variability and the first night effect [8].

Peripheral arterial tonometry (PAT) is a novel technique for HSAT and categorized as a Type 3 device together with respiratory polygraphy according to the American Academy of Sleep Medicine [7]. The PAT device is wrist worn and includes an accelerometer to detect movement. A finger probe measures the PAT signal and oxygen saturation. A chest sensor detects body position and includes a microphone to record an audio signal. This device setup has a low technical failure rate of 5.3% for at-home measurements [9].

In polysomnography, the cortical arousals from respiratory events are directly measured using electroencephalography. These cortical arousals also result in sympathetic activation and cause vasoconstriction mediated by alpha receptors. This vasoconstriction is measured in the finger to detect arousals of the autonomic nervous system. During these autonomic arousals, the PAT signal is attenuated, the heart rate increases, and the oxygen saturation decreases. Autonomic arousals are therefore a surrogate marker for cortical arousals when using electroencephalography [10].

PAT devices combine this information on arterial pulsatile arterial volume changes with heart rate variability, oxygen saturation, body position, and actigraphy to infer sleep-related breathing disturbances and sleep stages with their characteristic patterns. Since the PAT device does not measure airflow, all respiratory events are indirectly detected. To distinguish between indirectly and directly observed events, PAT events are referred to as peripheral arterial tonometry–derived apnea-hypopnea index (pAHI) and peripheral arterial tonometry–derived respiratory disturbance index (pRDI).

Previously, PAT recordings could only be analyzed using a proprietary, computer-based algorithm. This algorithm has been well validated for sleep-related breathing events and sleep stages [11, 12]. However, previously, no insight into the raw data has been possible. A novel software (zzzPAT® Itamar Medical, Caesarea, Israel) allows for manual scoring with visual oversight over the raw data of WatchPAT® recordings analogously to scoring for respiratory polygraphy or polysomnography. Zhang et al. developed an algorithm for manual oversight in an unselected patient cohort and could demonstrate that manual scoring improves the accuracy of sleep stages and respiratory events indices against polysomnography [10]. After the automated analysis is generated by the computer, the recordings are manually reviewed using visual oversight of the raw signals. First sleep stages and second respiratory events are classified by following an algorithm developed by Zhang et al. (further described in the “Methods”) [10].

To the knowledge of the authors, the manual algorithm has never been independently analyzed. It has been developed in an unselected patient collective and its effect on recordings performed at home and of patients with OSA is unclear. This article aims to analyze the clinical impact of manual scoring on sleep study results, such as pAHI or OSA severity classification. To answer this, we compared the results of automated and manual scoring and evaluated the clinical use of manual oversight of PAT recordings.

## Methods

For this study, we retrospectively reviewed data of patients who were referred for suspected obstructive sleep apnea to our ear, nose, and throat (ENT) clinic between 2017 and 2020 for further evaluation. The cohort was an unselected patient collective, which was referred to our ENT clinic specializing in sleep medicine because of suspected OSA. OSA was suspected by the referring colleagues either based on history with typical OSA symptoms, screening questionnaires, or pathological pulse oximetry. All recordings were performed as screening and diagnostic workup before treatment. Only recordings of patients who consented to the use of their data were included and the study has been approved by the local ethics committee. Basic anthropomorphic data, such as height, weight, age, and gender, were collected. All recordings were performed at home using the WatchPAT® 200 device (Itamar Medical, Caesarea, Israel). Recordings with less than 4 h of sleep time in the automated analysis were excluded from the analysis. The PAT recordings were automatically scored using the proprietary validated computer-based scoring algorithm for WatchPAT® scoring in the zzzPAT® software (Itamar Medical, Caesarea, Israel). The automated algorithm was configured with a cut-off value of 3% oxygen desaturation for respiratory events as recommended by the American Academy of Sleep Medicine [7]. The oxygen desaturation index (ODI) was calculated using the default setting of 4% desaturation, which cannot be changed by the user. Manual scoring was performed according to the novel guidelines for manual scoring of PAT with the zzzPAT® software (Itamar Medical, Caesarea, Israel) by an experienced sleep technician or the authors [10]. After the automated analysis was generated by the computer, sleep stages and respiratory events were reviewed by visual overview of the raw signals. Respiratory events were deleted if no reduction in the PAT signal with a corresponding increase in heart rate was observed or if they were associated with positional change. Events were also deleted if there was a desaturation of less than 3% and no snoring pattern changes were observed. Events were added if a reciprocal pattern of PAT signal reduction and heart rate increase was observed with a greater than 3% desaturation. In REM sleep, desaturations of greater than 4% were marked as events.

The data was analyzed for systematic differences between automated and manual scoring regarding pAHI, pRDI, and ODI over the whole night and in different sleep stages and body positions. Furthermore, the classification of OSA severity was compared between automated and manual scoring. OSA severity was classified with pAHI as no OSA <5/h, mild 5 ≤ 15/h, moderate 15 ≤ 30/h, and severe >30/h. Lastly, time and proportion of sleep in different sleep stages were compared. For positional OSA, the ratio between pAHI in supine and non-supine position was calculated (Cartwright index) [13]. The REM association, analogously, is the ratio between pAHI in REM and NREM sleep [14]. Oxygen saturation and time in different body positions were calculated, but no comparison of automated and manual scoring was performed since these parameters do not require manual editing. However, differences between automated and manual scoring may arise due to manual adjustment of sleep and wake times as well as individual sleep stages.

Since no simultaneous other recording methods, such as polysomnography, were performed, we compared the correlation of pAHI and ODI with both scoring methods. We used this correlation as a surrogate marker for improved accuracy because many publications find a linear relationship with ODI and AHI as well as ODI and RDI [15–17]. Since no simultaneous measurements were performed, this is a surrogate marker, and the focus of this article is the effect of manual scoring and not its accuracy.

Normal distributed data were analyzed using Student’s *t*-tests and ANOVA for multiple groups and for nonnormal distributed data Wilcoxon’s rank-sum test or Kruskal-Wallis’ test was used. Correlations were calculated using the Pearson correlation. RStudio (Boston, USA) was used for the statistical analysis. *P*-values below 0.05 were considered statistically significant.

## Results

PAT recordings from 130 patients were analyzed. The participants had a mean age of 53 ± 12 years and 72% (*n*=93) were male. The average body mass index was 27.6 ± 3.9 kg/m^2^ and the Epworth Sleepiness Scale was 7.9 ± 5.0. All recordings were automatically and manually scored and a comparison of both scoring methods is given in Table 1. Recording time, heart rate, and oxygen saturation do not require scoring and are therefore constant for both scoring methods.

**Table 1:** A comparison between automated and manual scoring. Recording time and parameters regarding oxygen saturation and body position are given only once since they do not require manual scoring. The oxygen desaturation index is not scored; however, differences arise due to adjustments in sleep time and sleep stages. pAHI refers to peripheral arterial tonometry–derived apnea-hypopnea index, pRDI to peripheral arterial tonometry–derived respiratory disturbance index, ODI to oxygen desaturation index, REM to rapid eye movement, and NREM to non-rapid eye movement. *P*-values are calculated using a two-sided Student’s *t*-test

	Automatic	Manual	*p*-value
Recording time (hours)	7.9 ± 1.2		
Sleep time (hours	6.7 ± 1.1	6.7 ± 1.1	0.87
pAHI total (events/hour)	27.5 ± 17.4	19.1 ± 15.2	<0.001
pAHI in REM sleep (events/hour)	32.8 ± 19.3	21.7 ± 16.7	<0.001
pAHI in NREM sleep (events/hour)	25.1 ± 17.7	17.5 ± 15.3	<0.001
pAHI in supine position (events/hour)	38.4 ± 25.1	30.0 ± 23.3	<0.01
pAHI in non-supine position (events/hour)	19.3 ± 15.8	11.4 ± 12.8	<0.001
Cartwright index (supine pAHI/non-supine pAHI)	4.2 ± 10.3	7.6 ± 26.4	0.19
REM association (REM pAHI/NREM pAHI)	1.7 ± 1.3	2.3 ± 5.3	0.24
pRDI total (events/hour)	31.2 ± 16.5	21.7 ± 14.4	<0.001
pRDI in REM sleep (events/hour)	36.2 ± 17.7	24.3 ± 15.8	<0.001
pRDI in NREM sleep (events/hour)	29.0 ± 17.3	20.2± 14.7	<0.001
pRDI in supine position (events/hour)	41.7 ± 23.7	33.5 ± 22.4	<0.01
pRDI in non-supine position (events/hour)	23.6 ± 15.5	15.5 ± 13.5	<0.001
ODI total (events/hour)	15.9 ± 14.5	15.8 ± 14.6	0.96
ODI in REM sleep (events/hour)	19.1 ± 16.5	19.1 ± 16.7	0.97
ODI in NREM sleep (events/hour)	14.1 ± 13.9	14.0 ± 14.0	0.93
ODI in supine position (events/hour)	24.8 ± 21.0	24.7 ± 21.1	0.97
ODI in non-supine position (events/hour)	8.8 ± 11.1	8.7 ± 11.1	0.89
Mean oxygen saturation (%)	94.0 ± 1.9		
Heart rate (beats/minute)	63.8 ± 8.3		
Time below 90% oxygen saturation (minutes)	15.6 ± 46.3		
Supine time (% of total sleep time)	45.3 ± 26.2	45.4 ± 26.1	0.96
REM sleep (% of total sleep time)	24.0 ± 7.1	23.6 ± 7.1	0.63
Light sleep (% of total sleep time)	60.1 ± 10.9	60.6 ± 11.0	0.75
Deep sleep (% of total sleep time)	15.8 ± 6.4	15.8 ± 6.3	0.99
Sleep time (% of total sleep time)	85.1 ± 6.1	85.3 ± 6.2	0.82
Wake time (% of total sleep time)	14.9 ± 6.1	14.8 ± 6.2	0.82

**Fig. 1:**
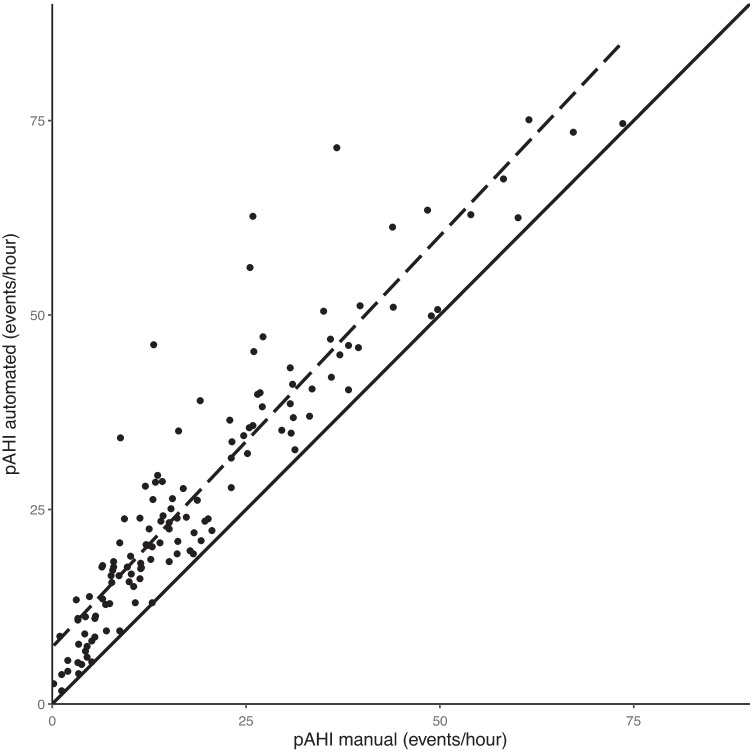
A scatter plot comparing peripheral arterial tonometry–derived apnea-hypopnea index (pAHI) for automated and manual scoring. The bold line is the line of perfect agreement between the two methods. A linear regression is given as a dashed line

**Fig. 2:**
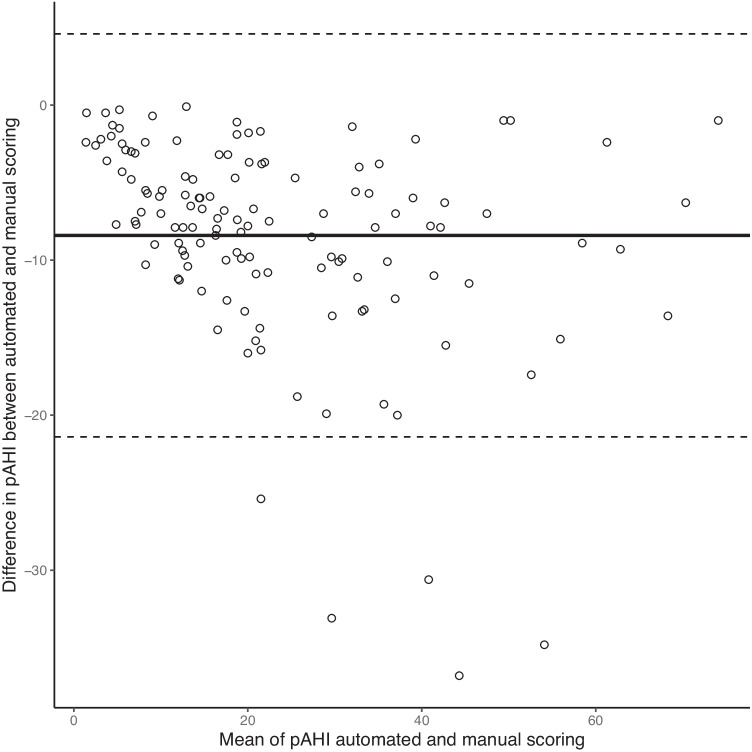
A Bland-Altman plot comparing automated and manual scoring of peripheral arterial tonometry–derived apnea-hypopnea index (pAHI). The bold line indicates the mean difference between automated and manual scoring, showing lower values for manual scoring (−8.4 events/h). The dashed lines indicate the lower and upper limit of agreement calculated as ±1.96 * the standard deviation

**Fig. 3:**
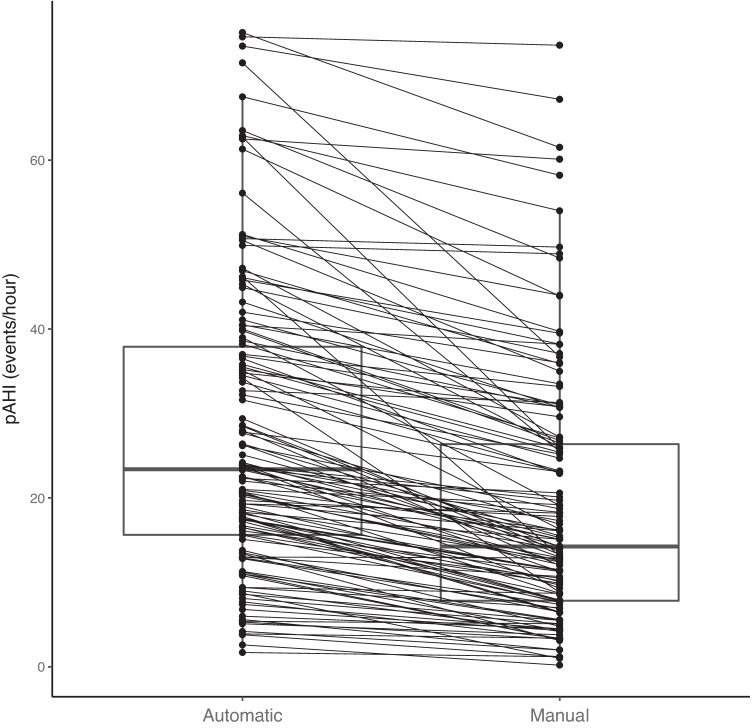
A comparison of automated and manual scoring. Lines connect the individual recordings illustrating a lower peripheral arterial tonometry–derived apnea-hypopnea index (pAHI) values for manual scoring

The mean pAHI of the whole night was 27.5 ± 17.4/h for automated scoring whereas it was only 19.1 ± 15.2/h (*p*<0.001) for manual scoring. The mean difference of pAHI between automated and manual scoring was 8.4/h. A direct comparison shows that the manually scored recordings lie almost exclusively below the automatically scored data for the whole range of OSA severity (see Fig. 1). The differences between automated and manual scoring are graphically illustrated with a Bland-Altman plot in Fig. 2 and a boxplot in Fig. 3. The lower results for manual scoring were consistent and significant in all sleep stages and body positions (see Table 1). With increasing sleep apnea severity, the absolute difference between automated and manual scoring increased (*p*<0.001), while the relative difference decreased (*p*<0.001). For patients with no OSA, the difference was −1.6h (−51%), for mild OSA −4.4/h (−44%), for moderate OSA −8.0/h (−38%), and for severe OSA −11.8/h (−26%). Similarly, a lower pRDI was observed for the manually edited data. The pRDI for the total sleep time was 31.2 ± 16.5/h and 21.7 ± 14.4/h (*p*<0.001), for automated and manual scoring, respectively.

Since ODI is calculated automatically and does not require scoring, differences between the scoring methods are a result of adjustments to the sleep and wake times and the individual sleep stages. Therefore, as expected only minimal, statistically not significant differences in ODI were observed with 15.9 ± 14.5/h for automated and 15.8 ± 13.5/h for manual scoring (*p*=0.96).

The analysis of pAHI, pRDI, and ODI depending on sleep stage and body position is given in Table 1. Manually scored pAHI and pRDI were significantly lower in all sleep stages and body positions compared to automatically scored data. Surprisingly, the positional OSA, given by the Cartwright index as the ratio between pAHI in supine and non-supine position, increased. The same was observed for REM-associated OSA, given by the ratio between pAHI in REM and NREM sleep, which also increased with manual scoring. These findings indicate that pAHI in non-supine position and NREM sleep decreased more than pAHI in supine and REM sleep.

In our cohort, men had a significantly higher pAHI than women with automated scoring of 30.3±17.2/h and 19.9±15.5/h (*p*<0.001) and manual scoring of 21.0±15.3/h and 13.9±13.9/h (*p*=0.003). Manual scoring reduced pAHI in men by −9.7/h (−34.7%) and women by −6.0/h (−36.2%). When accounting for the significantly higher pAHI in men, gender did not significantly influence scoring results (*p*=0.76). The body mass index category also showed no effect on scoring results (*p*=0.29, see Supplemental Fig. 1).

Both scoring methods resulted in a similar proportion for all sleep stages with no statistically significant difference. The REM sleep proportion was 24.0±7.1% and 23.6±7.1% (*p*=0.63) for automated and manual scoring, respectively.

**Fig. 4:**
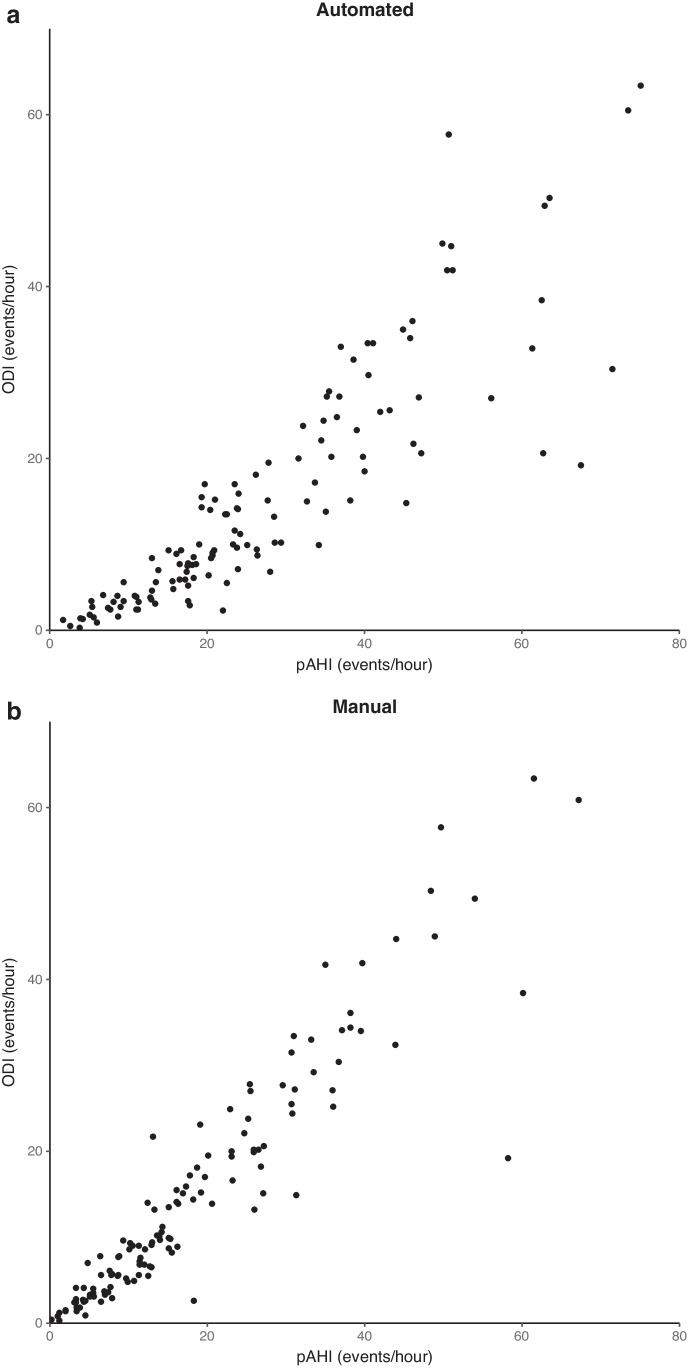
A correlation between peripheral arterial tonometry–derived apnea-hypopnea index (pAHI) and oxygen desaturation index (ODI) of automated (**a**) and manual scoring (**b**). The correlation significantly improves from 0.89 to 0.94 with manual oversight (*p*<0.001)

The Pearson’s correlation coefficient of pAHI and ODI increased from 0.88 for automated scoring to 0.94 for manual scoring (*p*<0.001, see Fig. 4). Similarly, the correlation of pRDI with ODI improved from 0.83 and 0.90, for automated and manual editing, respectively (*p*<0.001).

Manual editing lead frequently to changes in the OSA category. In only 64 recordings (49%), the category remained the same, whereas it decreased in 64 cases (49%) by one category and in 2 cases (2%) by two categories. No case of an increased OSA category was found.

## Discussion

PAT devices are increasingly used as an HSAT to diagnose OSA. Understanding the effect of the scoring method on results is important.

The automated algorithm is well validated and reproducible as well as time- and cost-efficient [11, 12]. The computerized algorithm is also objective, which makes it ideal for clinical studies by eliminating interrater variability. However, it has been demonstrated that this algorithm tends to overestimate respiratory events in patients with less severe OSA when compared to polysomnography [18]. A large cohort study of 500 patients undergoing simultaneous PAT and polysomnography found that PAT overestimated AHI by 4/h compared to polysomnography [19]. Yuceege et al. found AHI for PAT to be significantly higher than polysomnography with a mean difference of 1.78/h [20]. Both studies show an overestimation of respiratory events using PAT, but a lower difference than we observed between automated and manual scoring.

Manual scoring introduces some degree of subjectivity and variability in the analysis of sleep studies. Zhang et al. have developed a manual algorithm to improve the accuracy of both sleep stages and respiratory events [10]. In accordance with their results, we see manual scoring as clinically feasible requiring about 10–15 min to score one recording. The algorithm has been developed in an unselected patient collective, whereas our cohort consisted of patients with suspected sleep-disordered breathing [10].

Our study shows that sleep time and sleep stages are accurately recognized with the automated scoring algorithm of PAT. In the authors’ experience, the automated algorithm for PAT is very accurate in detecting sleep stages with little or no effect added by manual scoring.

However, there are significantly fewer respiratory events in the manually scored recordings. On average, the difference of pAHI was 8.4/h, which frequently resulted in a less severe OSA category. This difference is consistent among all OSA severities, both genders, and all body mass index categories. Zhang et al. found that manual scoring improved accuracy more in women than men [10]. Manual scoring is especially important in patients with mild or moderate OSA, for whom this difference can have implications for treatment recommendation and reimbursement from healthcare insurances.

Manual editing significantly improved the correlation of pAHI and pRDI with ODI in our study, indicating an improved accuracy since the correlation between AHI and ODI has been demonstrated in the literature [15–17].

Several limitations need to be mentioned. We did not perform simultaneous recordings with another measuring method, such as polysomnography. Without a direct comparison, it is impossible to describe the accuracy of either scoring method. We used the correlation of ODI with pAHI and pRDI as a surrogate marker for accuracy. This linear relationship has been demonstrated in many publications [15–17]. A large study by Ling et al. of more than 11,000 patients demonstrated an increasing ODI/AHI with body mass index [21]. However, since our patients had a narrow distribution of body mass index, we believe that the assumption of a linear relationship is appropriate. Furthermore, Zhang et al. have already demonstrated improved accuracy of manually edited PAT recordings compared to polysomnography in the development of the manual algorithm [10].

A further limitation of our study is that our patient collective was predominantly male and middle aged with a narrow range of body mass index. The recordings were performed in an unselected collective of patients with suspected OSA and retrospectively analyzed. We cannot further characterize our patients by comorbidities or detailed anthropomorphic measurements, because during the pretreatment process only incomplete data were collected. Moreover, the WatchPAT® 200 which was used for all recordings cannot differentiate between central and obstructive respiratory events.

A strength of this study is that all measurements were performed at home in the natural sleeping environment to reflect best the normal sleeping habits of the patients. Patients were instructed to follow their normal nighttime routine and abstain from influencing factors such as sleep medication or alcohol. However, these parameters were not recorded or controlled. To our knowledge, this is the first study to analyze the effect of manual PAT scoring for patients with suspected OSA and in recordings performed at home.

## Conclusions

The automated, computer-based algorithm offers a reliable, time- and cost-effective analysis of PAT recordings and eliminates interrater variability. Manual scoring allows for visual oversight over the recording assuring its quality. It also results in significantly lower respiratory event indices but does not significantly affect sleep time and sleep stages. We, therefore, conclude that manual scoring is important for respiratory events and to a lesser degree for sleep stages. Manual scoring might have a larger impact on patients with less severe OSA since treatment recommendations are more likely to change based on the manually scored data. Moreover, manual scoring can affect reimbursement (e.g., mandibular advancement devices), which may be dependent on cut-off values for AHI as it is common in most European countries.

Until improvements to the automated algorithm are implemented and validated, the authors recommend that sleep physicians decide individually if there is a need for manual scoring depending on the clinical situation and the possible impact on decision making.

## Supplementary Information

ESM 1 **Supplemental Fig. 1 Difference between automated and manual scoring in peripheral arterial tonometry-derived apnea-hypopnea index (pAHI) by gender (a) and by body mass index (b).** When accounting for sleep apnea severity, no statistically significant difference lies between the gender (p=0.76) or body mass index categories (p=0.29) (PNG 60 kb)

High Resolution Image (EPS 6 kb)

ESM 2 (PNG 67 kb)

High Resolution Image (EPS 7 kb)

## Abbreviations

AHI: apnea-hypopnea index
ENT: ear, nose, and throat
HSAT: home sleep apnea testing
NREM: non-rapid eye movement
ODI: oxygen desaturation index
OSA: obstructive sleep apnea
pAHI: peripheral arterial tonometry–derived apnea-hypopnea index
pRDI: peripheral arterial tonometry–derived respiratory disturbance index
PAT: peripheral arterial tonometry
RDI: respiratory disturbance index
REM: rapid eye movement
