# Supplementary figures and images for: Clinical impact of manual scoring of peripheral arterial tonometry in patients with sleep apnea
Source: Sleep Breath. 2022 Apr 2;27(1):229–37. doi: 10.1007/s11325-021-02531-9 (PMC9992081; doi:10.1007/s11325-021-02531-9)

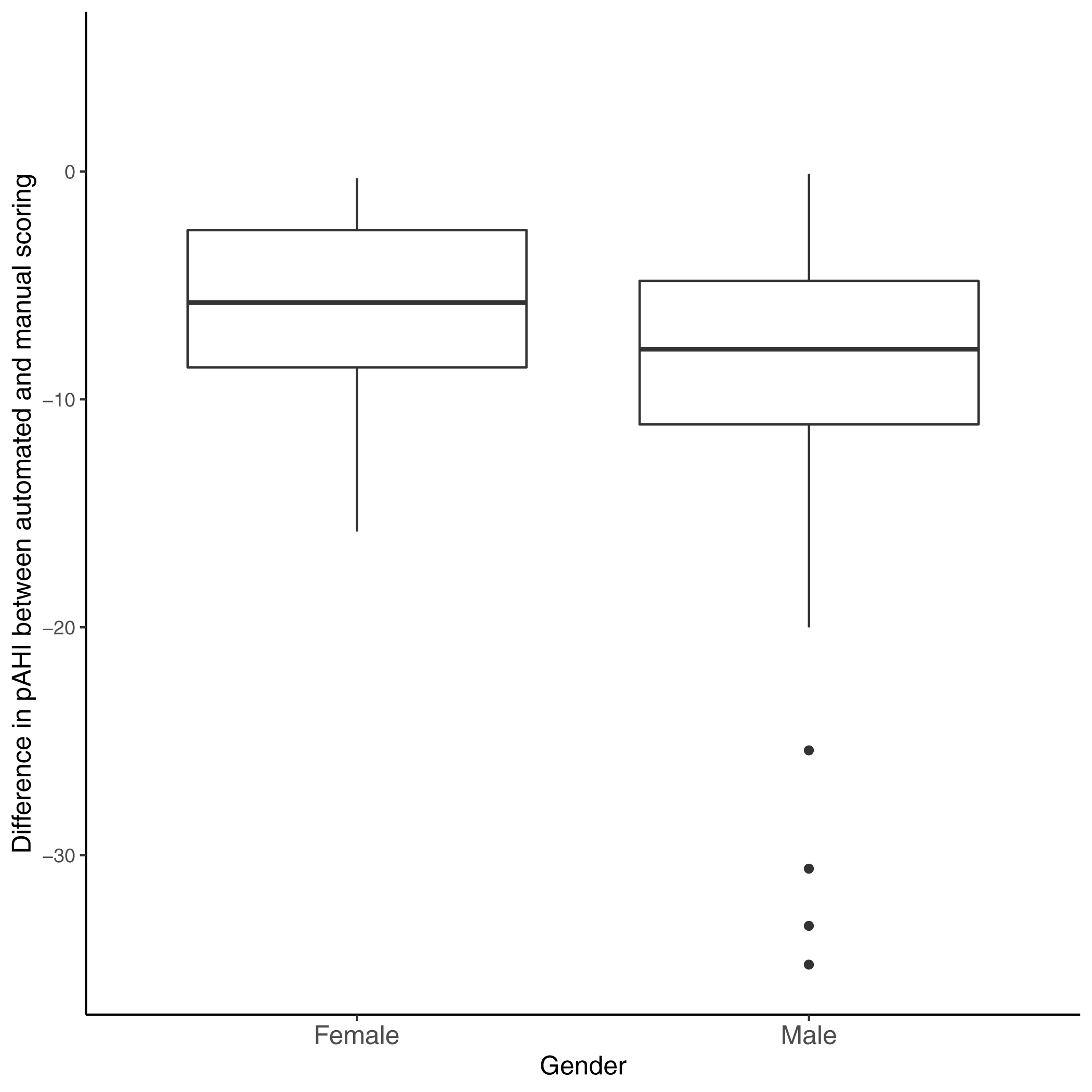

Supplement: Supplementary file 1 — Supplemental Fig. 1 Difference between automated and manual scoring in peripheral arterial tonometry-derived apnea-hypopnea index (pAHI) by gender (a) and by body mass index (b). When accounting for sleep apnea severity, no statistically significant difference lies between the gender (p=0.76) or body mass index categories (p=0.29) (PNG 60 kb) [file 11325_2021_2531_Fig5_ESM.png]

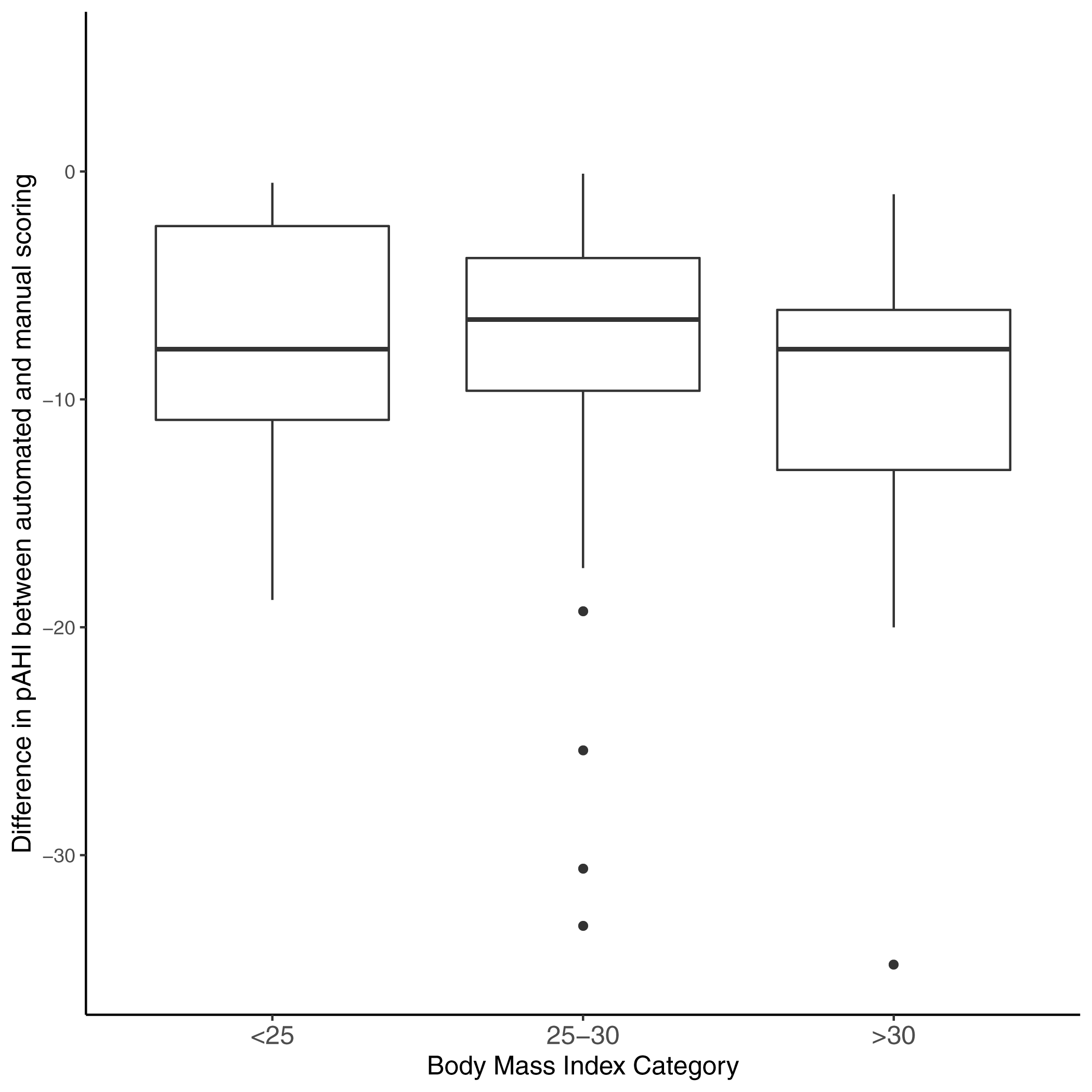

Supplement: Supplementary file 3 — (PNG 67 kb) [file 11325_2021_2531_Fig6_ESM.png]
